# Three-dimensional assessment of internal adaptation measurement of three cad/cam ceramic systems

**DOI:** 10.1590/0103-6440202204981

**Published:** 2022-12-05

**Authors:** Emad M Elsharkawy, Ahmed MY ElKouedi, Tamer E. Shokry

**Affiliations:** 1 Fixed Prosthodontics Department, Faculty of Dental Medicine, Boys, Cairo, Al-Azhar University, Egypt.

**Keywords:** internal fit, nanoceramics, hybrid ceramics, triple-scan protocol

## Abstract

This study aimed to evaluate the internal adaptation of three different computer-aided design/computer-aided manufacturing (CAD/CAM) ceramic crowns. The internal adaptation of a polymer-infiltrated ceramic network material (Vita Enamic [VE]) was compared to two machinable glass-ceramics; Zirconia-reinforced lithium silicate (Vita Suprinity [VS]) and a lithium disilicate glass-ceramic (IPS e.max. CAD). Thirty human premolars of average size were prepared (n=10 each group) by computer numerical control to fulfill the criteria of all-ceramic crown design. Optical impressions were taken for each tooth preparation using the CAD/CAM scanner. Thirty crowns were fabricated using CAD/CAM system and divided into three groups (IPS e.max, VE, and VS). To assess the internal fit of tested crowns, the gap between the intaglio of each crown and the corresponding tooth surface was evaluated using a 3D digital scanner using the Triple-scan Protocol. One-way ANOVA followed by Tukey Post Hoc statistical tests were used to statistically analyze results of the internal fit. There was a statistically significant difference for all groups at the four axial walls (p = 0.000002). For total internal fit between groups, comparisons showed a statistically significant difference between all tested groups (p=0.000002). When each pair of groups was statistically compared with each other, all pair comparisons showed a statistically significant difference. IPS e.max CAD had the best internal fit, followed by Vita Enamic, then Vita Suprinity. For all ceramics tested, values of internal fit of all ceramics tested were within the clinically acceptable range.

## Resumo

Este estudo teve como objetivo avaliar a adaptação interna de três diferentes coroas cerâmicas CAD/CAM (Computer-aided design/computer-aided manufacturing). A adaptação interna de um material cerâmico de rede com infiltração de polímero (Vita Enamic [VE]) foi comparada com duas cerâmicas de vidro maquináveis; silicato de lítio reforçado com zircónio (Vita Suprinity [VS]) e uma cerâmica-vidro de dissilicato de lítio (IPS e.max. CAD). Trinta dentes pré-molares humanos de tamanho médio foram preparados (n=10 cada grupo) por controle numérico computadorizado para preencher os critérios de desenho de coroa em cerâmica pura. Foram tiradas impressões ópticas para cada preparação dentária utilizando o scanner CAD/CAM. Trinta coroas foram fabricadas usando o sistema CAD/CAM e divididas em três grupos (IPS e.max, VE, e VS). Para avaliar o ajuste interno das coroas testadas, o espaço entre o entalhe de cada coroa e a superfície dentária correspondente foi avaliado usando um scanner digital 3D usando o Protocolo Triple-scan. Para analisar estatisticamente os resultados do ajuste interno, utilizou-se a ANOVA unidirecional seguida dos testes estatísticos Tukey Post Hoc. Houve uma diferença estatisticamente significativa para todos os grupos nas quatro paredes axiais (p = 0,000002). Para o ajuste interno total entre grupos, as comparações mostraram uma diferença estatisticamente significativa entre todos os grupos testados (p=0,000002). Quando cada par de grupos foi comparado estatisticamente uns com os outros, todas as comparações de pares mostraram uma diferença estatisticamente significativa. IPS e.max CAD teve o melhor ajuste interno, seguido por Vita Enamic, depois Vita Suprinity. Para todas as cerâmicas testadas, os valores de ajuste interno de todas as cerâmicas testadas encontravam-se dentro da gama clinicamente aceitável.

## Introduction

A dental restoration's internal and marginal adaptation to the abutment is critical for long-term clinical success. Microleakage, plaque accumulation, gingival irritation, and recurrent caries are all increased by inadequate marginal adaptation^1^. Furthermore, a large internal gap will affect the restoration's fracture resistance^2^. The use of computer-aided design and computer-aided manufacturing (CAD/CAM) technology for indirect restorations has been a significant shift in restorative dentistry in recent decades. This CAD/CAM method has three phases: digitalization of the tooth preparation, virtual restoration design with a computer, and restoration production^3^. The CAD/CAM technique, design, and milling stages would reduce production costs by lowering technician time in the lab, allowing prosthodontists to construct a restoration in the office^4^. Intraoral Scanning plus digital milling may increase restorative adaptability^5^. Moreover, CAD/CAM technology allows restoring both anterior and posterior teeth using a wide selection of aesthetic dental materials^6^.

The laboratory adaptation of conventionally fabricated all-ceramic restorations is a delicate technique that can be influenced by a variety of factors, including impression material and technique, disinfection, storage time, and impression conditions before pouring the stone cast, application of the die spacer, and the investment and casting or pressing process^7^. In CAD/CAM technology, additional inherent aspects affect the adaption of milled restorations, including scanner accuracy, software design, spacer configuration, milling unit precision, and milled material qualities^8^. However, CAD/CAM systems use highly precise scanners, recent software, and accurate milling devices with developed technology ^9^ ^-^ ^11^.

A wide variety of aesthetic CAD/CAM ceramics, ranging from slight weak feldspathic and reinforced glass ceramics to high-strength lithium disilicate glass ceramics, zirconia, and hybrid ceramics, has recently been introduced^12^. Some of these mechanical properties have been examined. However, there is little data in the literature about the marginal gap and internal adaptations of CAD/CAM ceramic materials; additionally, such studies have been restricted to zirconia and lithium disilicate^13^. The marginal gap and internal adaptations of CAD/CAM lithium disilicate crowns were equivalent or better than conventionally fabricated crowns^13^ ^,^ ^14^.

Pre-crystallized blocks of lithium disilicate are available to assist in machining during the CAM phase. Following milling, the restorations go through a crystallization process to reach their maximum strength and develop the color and translucency of enamel^15^ ^,^ ^16^. Zirconia reinforced lithium silicate glass-ceramic (ZLS) restorations have been introduced to the dental market. This glass-ceramic is loaded with zirconia particles (10% by weight) to merge the advantages of glass-ceramic translucency with the mechanical properties of zirconia. It is claimed that incorporating zirconia particles improves mechanical properties by preventing crack propagation^17^ ^,^ ^18^.

The hybrid dental ceramic has a dual-network structure that merges the best properties of ceramic and composite materials. The ceramic network has about 86% share of the material and is reinforced by a polymer network^19^. It has a long and illustrious history of directly observing the marginal adaptation under a microscope or making measurements on cross-sections after sectioning the cemented restoration. Various methods for assessing restoration adaptation have been developed. Microcomputed tomography can be used for 3D adaptation evaluation^20^. Holst et al.^21^ recently described a triple-scan protocol for 3D adaptation assessment of dental restorations in which the restoration, abutment, and assembly are digitized and aligned in preparation for analysis. The literature is still unclear about the internal fit of different dental ceramics to the underlying teeth prepared, so, this study aimed at investigating the internal fit of three different CAD/CAM ceramics. The hypothesis of this study was that there will be a difference in the internal adaptation of the three tested ceramic materials.

## Material and methods

The ethics committee at Al-Azhar University's Faculty of Dental Medicine approved this study with approval number (758/2595). Natural premolar teeth of average size (7.89±1 mm buccolingually and 6.98±1 mm mesiodistally) were prepared by computer numerical control (CNC) to fulfill the criteria of all-ceramic crown preparation design for use in the study and divided into three main groups: IPS. Emax CAD (EC), Vita Suprinity (VS), and Vita Enamic (VE), ten crowns each.

Thirty freshly extracted human maxillary first premolar teeth were collected from the orthodontic department of Al-Azhar University. The sample size (n=10) in each group was calculated to have a 80% power to detect a difference between means with a significance level (alpha) of 0.05 (two-tailed) using GraphPad StatMate 2.00.

Selection criteria were based on teeth condition and average size measured by a digital caliper. The buccolingual diameter of the selected teeth equals 7.89±1ml, and the mesiodistal width equals 6.98±1ml. Teeth were randomly distributed into three groups, ten teeth in each one. A Parallometer (BEGO, Paraflex, Germany) was used to allow proper orientation and vertical centralization of the tooth inside the plastic PVC mold.

To standardize the preparation dimensions, a CNC (Premium 4820, imes-icore, Eiterfeld, Germany) four-axis milling machine was used on teeth preparation. Using a diamond endmill under oily water coolant, the CNC machine was adjusted to reduce all teeth to fulfill the criteria of all-ceramic crown design (1-mm rounded shoulder finish line, 2.0 mm occlusal surface reduction, and 6 degrees of axial convergence angle).

Optical impressions were taken by Scanning and digitizing the dies using the 3Shape D700 dental lab scanner (3Shape A/S, Holmens Kanal 7, 1060 Copenhagen K Denmark). Crown was selected as the restoration type with the design mode set to biogeneric (maxillary first premolar). Restoration parameters were set, including the spacer thickness at 60 μm, and all other parameters were kept according to the software default. The insertion axis was determined to avoid any undercuts, as the incorrect insertion axis may result in a thin or even a perforated wall. A total of 30 crown specimens (10 from each material) were fabricated from optical data using a 5-axis (CAD/CAM) milling machine (WIELAND Zenotec, Ivoclar Vivadent, Germany). Specimens were placed on their corresponding prepared teeth, and the seating of each crown was evaluated using a magnification loupe (5X) to perform an initial clinical evaluation. The IPS. Emax CAD and Vita Suprinity specimens for the final crystallization cycle were positioned in a ceramic furnace (The programat P310 furnace; Ivoclar Vivadent, Germany). No extra polishing or finishing was needed for the Vita Enamic.

The gap between the intaglio of each crown and the corresponding tooth surface was measured using a 3D digital scanner to assess the internal fit of the tested crowns. The master die and the intaglio of each of the crowns were digitized using MEDIT i500 (MEDIT Corp, Korea), which is a powder-free 3D intraoral scanner.

Three different scans were made according to the triple-scan protocol by Holst et al.^21^. The first scan was taken to digitize the prepared tooth. This was achieved by capturing the tooth prepared as an upper jaw scan. The second scan was taken to digitize the outer and intaglio surfaces of each of the crowns. The third scan was taken to digitize each crown while seated on its corresponding tooth in its correct final position. An occlusion scan was selected for this step. After processing the scans, STL files were exported and sent to 3D inspection software ( Poly Works Innovmetric 2007, Canada) for fit analysis and 3D color mapping. Firstly, the STL file of the die and the crown-die STL file were registered by manual alignment (pre-alignment using point pairs) followed by best-fit registration of the area not covered by the crown. A suitable number of point pairs on the software were automatically picked. The same procedures were repeated for the alignment of the crown and crown-die STL files for all samples.

The fit assessment was done by making a 3D coloring map. The red color indicates optimum fit, which decreases as the color turns green. For the 3D measurements, two sections, one buccolingual and one mesiodistal were made through aligned data figure ^(^ ^1^ ^-^ ^2^ ^)^. The distance between the die and the intaglio surface of the crown was measured at six standardized points (2 on the margins, two on the mid axial walls, and two on the occlusal surface). The final data obtained were exported, collected, tabulated, and then statistically analyzed.

**Figure 1:**
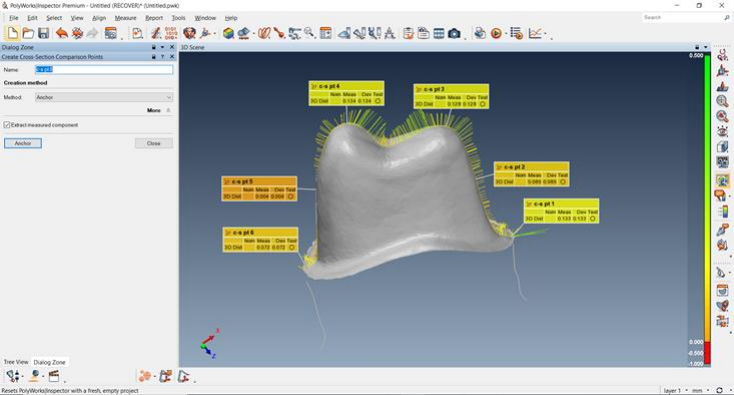
Bucco-lingual section was made through aligned data

**Figure 2:**
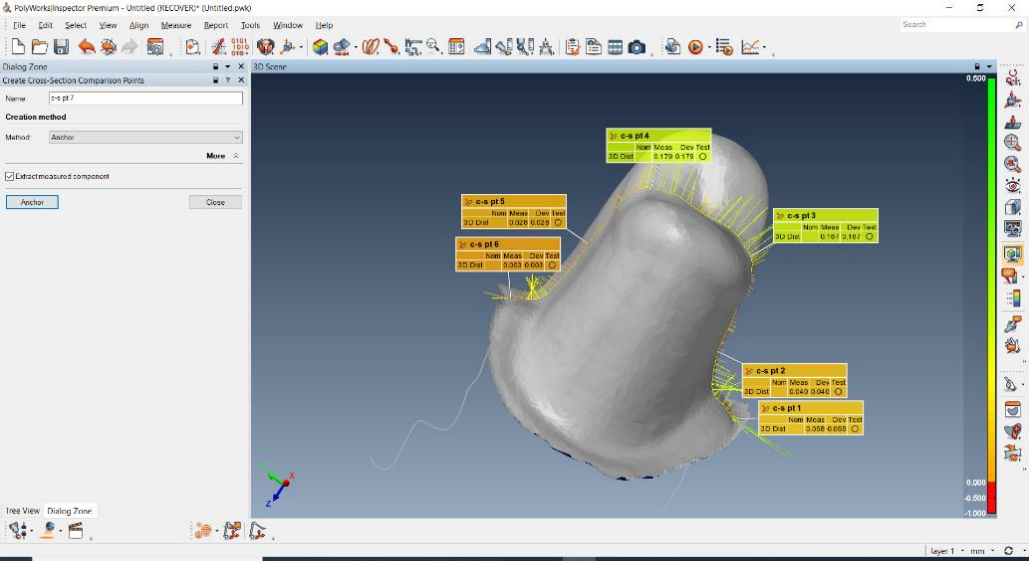
Mesio-distal section was made through aligned data.

Data were represented by the mean, standard deviation (±SD), with 95% Confidence Interval (95% CI) values. One-way ANOVA, Tuckey's post hoc, and descriptive statistics were used to compare different materials. Statistical analysis was performed with IBM SPSS Statistics version 20 at a 95% confidence interval (The significance level was set to P ≤ 0,05).

## Results

Statistical analysis was set to compare the four axial walls. The measurement of each wall was recorded from a calculation of an average of all readings recorded. Each wall was measured at 3 points (marginal, mid-wall, and occlusal points). For each section made in both the mesiodistal and buccolingual directions, 6 points were taken as a reference for measurements.

There was a statistically significant difference in the internal fit of the three tested groups (p = 0.000002*). For all groups, the internal fit of the distal wall was statistically significantly different than that of the average of measured points of the mesial, buccal, and lingual walls of corresponding samples.

For e.max CAD, there was no statistically significant difference when the Mesial, Lingual and Buccal walls were compared (p=0.067). Meanwhile, a statistically significant difference was recorded when each of the three walls was compared to the Distal wall (M and D, p=0.0004*), (B and D p=0.0015*), and (L and D p=0.00052*).

For Vita Enamic, there was no statistically significant difference when the Mesial, Lingual and Buccal walls were compared (p=0.05). Meanwhile, a statistically significant difference was recorded when each of the three walls was compared to the Distal wall (M and D, p=0.00013*), (B and D p=0.0023*), and (L and D p=0.00021*).

**Figure 3:**
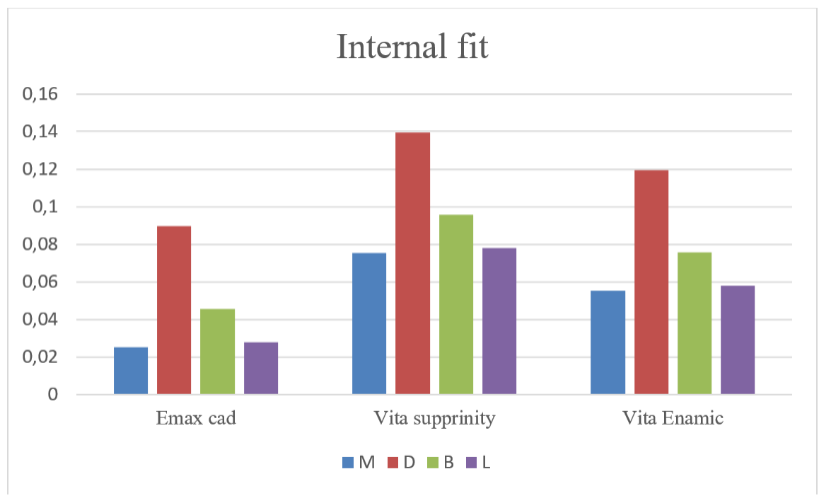
A column chart showing a comparison of the internal fit to each wall (M=Mesial, D= Distal, B= Buccal, and L= Lingual) of each group for the three tested materials.

For Vita Suprinity, there was no statistically significant difference when the Mesial, Lingual and Buccal walls were compared (p=0.05). Meanwhile, a statistically significant difference was recorded when each of the three walls was compared to the Distal wall (M and D, p=0.00015*), (B and D p=0.0026*), and (L and D p=0.00033*). All data are represented in table 1 and figure 3.

For total internal fit, there was a statistically significant difference between all tested groups (p=0.000002*) table 2. When each pair of groups was statistically compared with each other, all pair comparisons showed a statistically significant difference; EC and VS (p=0.000003*), EC and VE (p=0.0008*), VS and VE (p=0.016*). e.max CAD had the best internal fit, followed by Vita Enamic, then Vita Suprinity.

When the mesial wall was compared to its counterpart of the other two groups, there was a statistically significant difference (p=0.000001*). Pair comparisons between each set of materials showed a statistically significant difference; EC and VS (p=0.00000002*), EC and VE (p=0.000004*), VS and VE (p=0.0004*). e.max CAD had the best internal fit, followed by Vita Enamic, then Vita Suprinity.

When the distal wall was compared to its counterpart of the other two groups, there was a statistically significant difference between the three groups (p=0.038*). Pair comparisons between each set of materials showed a statistically significant difference between EC and VS (p=0.014*), whereas there was no statistically significant difference between comparisons of the other pairs; EC and VE (p=0.12), VS and VE (p=0.29). E.max CAD had the best internal fit, followed by Vita Enamic, then Vita Suprinity.

**Table 1:** Comparisons of the results of internal fit between the four axial walls within the same ceramic group.

		The internal fit between groups (μm)			P-value
		EC	VS	VE	
B	Mean	0.045598^Aa^	0.095598^Ba^	0.075598^Ca^	0.00003*
	±SD	0.020402	0.018408	0.040667	
L	Mean	0.028079^Aa^	0.078079^Ba^	0.058079^Ca^	0.000004*
	±SD	0.015539	0.028943	0.040651	
M	Mean	0.025237^Aa^	0.075237^Ba^	0.055237^Ca^	0.0000001*
	±SD	0.010401	0.02746	0.022465	
D	Mean	0.089573^Ab^	0.139573^Bb^	0.119573^Cb^	0.0386648*
	±SD	0.041495	0.024151	0.027607	

*Significant difference p≤0.05. Different UPPERCASE letters indicate statistical significance between different ceramic groups, whereas different lowercase letters indicate statistical significance between axial walls within the same ceramic group.

**Table 2:** Comparisons of internal fit between groups.

		The Internal fit between groups (μm)			P value
		EC	VS	VE	
Total	Mean	0.047122 ^A^	0.097122 ^B^	0.077122 ^C^	0.000002*
	±SD	0.0169	0.0169	0.0169	

* Significant difference p≤0.05

When the buccal wall was compared to its counterpart of the other two groups, there was a statistically significant difference (p=0.00003*). Pair comparisons between each set of materials showed a statistically significant difference; EC and VS (p=0.00003*), EC and VE (p=0.004*), VS and VE (p=0.0417*). E E.max CAD had the best internal fit, followed by Vita Enamic, then Vita Suprinity.

**Figure 4:**
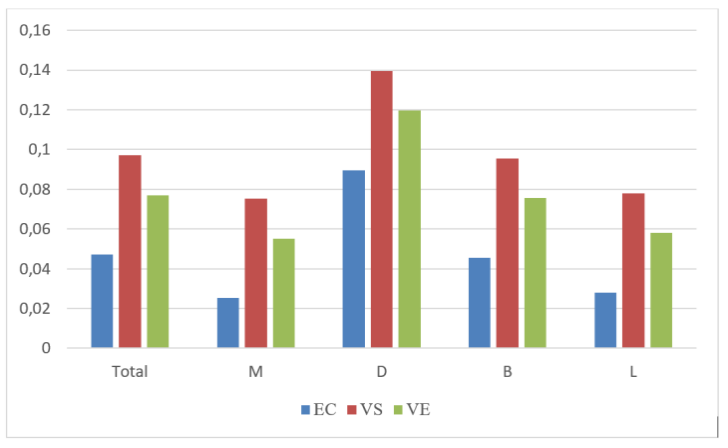
A column chart showing comparisons of internal fit between groups.

When the lingual wall was compared to its counterpart of the other two groups, there was a statistically significant difference (p=0.000004*). Pair comparisons between each set of materials showed a statistically significant difference; EC and VS (p=0.000001*), EC and VE (p=0.0004*), VS and VE (p=0.011*). E E.max CAD had the best internal fit, followed by Vita Enamic, then Vita Suprinity. All data are represented in table 1 and figure 4 .

## Discussion

The adaptation between a tooth and a restoration is critical to the long-term success of dental restorations^1^ ^,^ ^4^. As a result, an effort has been made to standardize the process of dental treatment, from abutment tooth preparation to crown milling and final insertion and cementation. The purpose of this study was to evaluate the internal adaptation of various CAD/CAM ceramic crowns using the triple-scan method. The triple-scan method allows for a comprehensive 3D adaptation assessment, with the cement-gap thickness measured at almost any position. This is useful because, in most restorations, the gap after cementing is not evenly distributed^23^. A CNC was used to perform a standard tooth preparation. As a result, the effects of preparation geometry and margin design on crown adaptation could be eliminated^5^.

According to the results of the current study, there is a significant difference in the internal fit of the three ceramic materials. When each pair of groups was statistically compared to each other, all pair comparisons showed a statistically significant difference, e.max CAD had the best internal fit, followed by Vita Enamic, then Vita Suprinity.

These findings can be explained in terms of the virtual cement space created by computer software. The cement space is primarily intended to create an internal gap between a restoration's fitting surface and underlying tooth preparation. This not only makes room for the cement to be used, but it also helps to prevent any interference with the seating of the fixed dental restoration. While the cement space measurement is known, the exact volume of that space is unknown. The cement space volume is a three-dimensional measurement that simply represents a more accurate value than a two-dimensional measurement. Kim et al.^24^ determined the cement space volume of IPS e.max CAD molar crowns using various scanners and reported a range of 25.3 to 40.7 mm. This variation may exist not only between different restorations but also between different parts of the fitting surface of the same restoration.

In the present study, the cement space was designed digitally for the CAD/CAM restorations during the computerized design process. However, the question remains as to whether the CAD/CAM devices can transfer the cement space range from the milling unit to the restoration with the same precision. Milling unit parameters such as bur use times, milling material, and drill diameter, as well as tooth preparation factors such as preparation method, anatomic conditions, occlusal preparation, cervical line curvature, and axial angle, can all have an impact on the final fit of the intaglio to the underlying preparation^25^. The ability of the CAD/CAM device to process the details of the restorations is inversely proportional to the diameter of the rotary instruments. The small-diameter rotary instruments allows a finer detail, however they have reduced endurance and higher production cost^7^.

The three ceramics used in this study have a unique composition for each. Vita Enamic (VE) is a hybrid ceramic with resin content infiltrating the ceramic network. This resilient resin component affects the material properties, not only in its final clinical performance but also during restoration manufacturing. On the other hand, the other two ceramics, IPS e.max CAD (EC) and Vita Suprinity (VS), are two glass-ceramics. The difference in elastic properties between the VE on the one hand and VS and EC, on the other hand, could be attributed to the resin component, which helps to reduce brittleness. Unlike EC and VS, which retain their crystal structure and rupture during the milling process, VE does not require a crystallization phase. This distinguishing feature may have affected the results. Although both EC and VS have a glassy matrix in their composition, VS has the unique feature of zirconia particles added to the lithium silicate glass ceramic as means of strengthening, making it, unlike the EC in its final composition.

Mously et al.^22^ reported that marginal and internal adaptations of CAD/ CAM fabricated restorations may have been affected by several factors, including fabrication technique, preparation design, spacer thickness, the scanning method and its accuracy, the software, the restorative material, and the properties of milling machine. Although Vita Enamic, Vita Suprinity, and IPS. Emax CAD crowns showed significant differences in internal fit, and the values were within the clinically acceptable range, IPS. Emax CAD had the best internal fit, followed by Vita Enamic and Vita Suprinity. Based on the findings of this study, clinicians can use the CAD/CAM fabrication of any of the tested ceramics that can give satisfactory internal adaptation. This in turn, may aid in the long-term success of a final restoration. A limitation of the current study is that only a single spacer thickness (60μm) has been evaluated. Further studies are needed in which different spacer thicknesses are used. According to the results of the current study, it can be concluded that IPS e.max CAD is superior to Vita Enamic and Vita Suprinity in terms of internal adaptation.
